# Self-blame Attributions of Patients: a Systematic Review Study

**DOI:** 10.5195/cajgh.2020.419

**Published:** 2020-03-31

**Authors:** Yadollah Jannati, Hamid Sharif Nia, Erika Sivarajan Froelicher, Amir Hossein Goudarzian, Ameneh Yaghoobzadeh

**Affiliations:** 1 Psychiatry and Behavioral Sciences Research Center, Mazandaran University of Medical Sciences, Sari, Iran; 2 Amol School of Nursing and Midwifery, Mazandaran University of Medical Sciences, Sari, Iran; 3 Department of Physiological Nursing, School of Nursing, University of California San Francisco, San Francisco, California, USA; 4 Department of Epidemiology & Biostatistics, School of Medicine, University of California San Francisco, San Francisco, California, USA; 5 Student Research Committee, Mazandaran University of Medical Sciences, Sari, Iran; 6 School of Nursing and Midwifery, Tehran University of Medical Sciences, Tehran, Iran

**Keywords:** Self-blame, Patient, Anxiety, Depression, Systematic review

## Abstract

**Introduction:**

Psychological aspects are important issues in patients that will have significant effects on disease progression. A new and important psychological concern is self-blame. This review was performed with the aim of systematic review on studies around patient’s self-blame.

**Methods:**

This is a systematic review using international databases including PubMed (since 1950), Scopus (since 2004), Web ofSciences (since 1900), and ProQuest (since 1938) and Irani an databases including SID (since 2004) and Magiran (since 2001). Mesh terms including “patient,” “regret,” and “guilt” and non-Mesh terms including “self-blame attribution,” “characterological self-blame,” “behavioral self-blame,” and “blame” were used in Iranian and international databases with OR and AND operators.

**Results:**

The review yielded 59 articles; 15 articles were included in the present study. The ages of patients ranged from 29-68.4 years. Most of studies (86.6%) had cross-sectional design and use characterological self-blame and behavioral self-blame variablesfor assessing self-blame attributions. The results showed that in most studies, a significant relationship among self-blame and psychological distress, anxiety, and depression were reported.

**Conclusions:**

A significant relation was reported between self-blaming and the degree of distress, anxiety, and depression in patients in most of the studies.

Vos et al. estimated about 95% of the world's populations are suffering from a variety of physicalhealth problems^1^. Cancer, cardiovascular disease, chronic obstructive pulmonary disease (COPD), and diabetes are the most common health problems in the world. Though at a lower incidence, populations also suffer from diseases such as acquired immunodeficiency syndrome (AIDS), kidney disease, and hypertension^2^. These are chronic conditions that typically require long-Amol School of Nursing and Midwifery, Mazandaran Univ ersity of Medical Sciences, Sari, Iran;^3^ term care throughout life and often have many physical, psychological, and social challenges for the patient and their families^3^.

Psychological aspects are some of the most important problems these patients experience, and they have significant effects on the progression of their disease^4^. The psychological adaptation to these stressful life events needs to be assessed, including cognitive effects. Cognitive assessments are important in determining these effects, explored/identified through factors like self-blame^5^. Self-blame is a common reaction to stressful events and has certain effects on how individuals adapt. Types of self-blame are hypothesized to contribute to depression, and self-blame is a component of self-directed emotions like self-guilt and self-disgust. Because of self-blame's commonality in response to stress and its role in emotion, self-blame should be examined using psychology's perspectives on stress and coping ^5^,^6^. Janoff-Bulman suggested that behavioral self-blaming has a direct relation with the level of health and the ability to control stress. Because these chronic conditions persist over many years and are often unstable, they have a damaging effect on psychological well-being^6^. Many studies with patient samples other than cardiovascular disease confirmed the hypothesis that behavioral self-blaming is directly related to better health findings than characterological self-blaming^5^,^7^,^8^.

Numerous other studies have been conducted about self-blaming in patients^5^,^9^^-^^12^; though some contradictions are still presented. For example, the relationship between behavioral self-blaming and characterological self-blaming and psychological disorders was investigated in a study on cardiovascular patients receiving cardiac rehabilitation exercises^12^. The results indicated that behavioral self-blaming had a positive effect on the level of anxiety and depression at the beginning of exercise. Characterological self-blaming, however, did not have any definite relationship with these variables. Both types of self-blaming were good predictors of cardiac psychological symptoms in patients with cardiac rehabilitation in other studies^10^. The results of this study showed that both types of self-blaming initially had a positive relationship with the symptoms of heart disease, but only characterological self-blaming was a predictor of outcomes in the ensuing 21 months.

Therefore, studies on self-blaming of patients needed to be made comprehensively by means of an instrument that emphasizes both categories of blaming. Considering the contradictory information across studies it seems necessary to do a review of all available literature on this topic to better understand the relationship between self-blaming and psychological disorders in different populations. Therefore, considering the necessity of a comprehensive review, the present study was conducted with the aim of systemically reviewing studies in the field of self-blaming in patients, with the intent of exploring if self-blame is associated with any particular type of medical conditions.

## Methods

This systematic review was conducted in accordance with PRISMA guidelines 2019^13^. This study reports on a systematic review of studies in the field of self-blaming.

### Search strategy and studies' criteria

A systematic review of related studies conducted in the international databases in the English and Farsi literature of Pub Med (since 1950), Scopus (since 2004), Web of Sciences (since 1900), and ProQuest (since 1938) and Iranian databases of SID (since 2004) and Magiran (since 2001). Based on table 1, Mesh terms including “patient”, “regre “guilt” and non-Mesh terms including “self-blame attribution”, “characterological self - blame”, “behavioral self - blame” and “blame” were chosen to search using OR and AND operators in Iranian and international databases, respectively. All original articles were collected and reviewed for inclusion in the study. First, the published articles related to the aim of the present study were reviewed by the two researchers (AHG and HSH). The initial list was then entered into Endnote to eliminate duplicate studies if present. In the third stage, the articles were examined and filtered based on inclusion and exclusion criteria. Inclusion criteria were: 1) the key words (including self-blame, characterological self-blame, behavioral self-blame, and blame) presented in title or abstract; 2) published in Persian or English language; 3) self-blaming considered as the independent variable; 4) only subjects with a physical diagnosis were included (due to the fact that there are differences between these patients and the ones with mental disorders, which makes it difficult to reach accurate conclusions). Published studies in the form of dissertations, books, and abstracts of congresses and conferences were also excluded from the study, due to insufficient details and lack of peer review. Review studies were also excluded from the study. Also, articles with access limitation to the full text were received in correspondence with the author.

**Table 1.** Database search stages

Databases	Search strategy	Preliminary searches	Formal screening of search results against eligibility criteria
PubMed		9,704	9
Scopus		2,365	3
Web of Science	[(Self-blame attribution) OR Blame] OR [Regret OR Guilt) AND Patient	3,921	2
ProQuest		91,955	1
SID		67	0
Magiran		280	0

**Figure 1.**
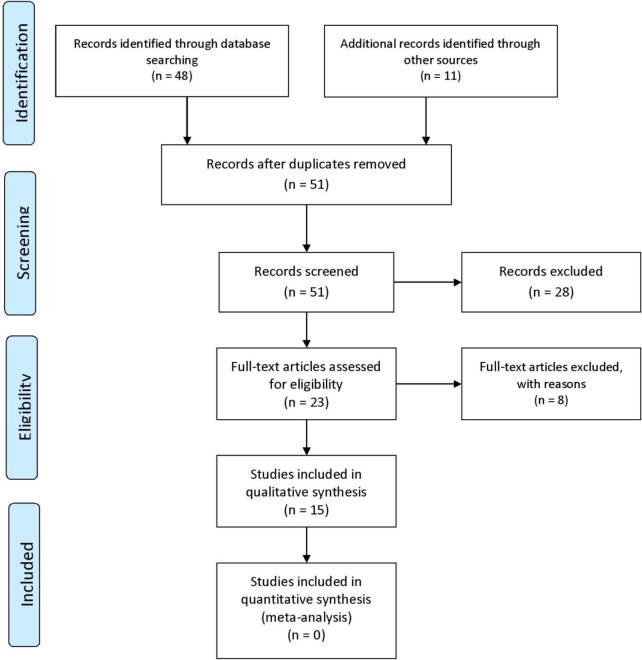
PRISMA flowchart

Based on Table 1 and Figure 1, from 108,292 articles that were found from all of databases, 51 articles were extracted. After across mentioned criteria, 36 articles were excluded. Then, 15 articles were entered to quality assessment stage.

### Quality assessment

The quality of the methodology was verified by two researchers (AHG and YJ) independently using the STROBE checklist (for cross-sectional studies) and the COREQ checklist (for qualitative studies). STROBE consists of 22 sections and examines various aspects of the methodology, including the objectives of the study, sampling methods, variables measurements, statistical analysis, confounding modifications, and validity and reliability of the measures^14^. The minimum score was considered as 16 for this checklist. Finally, qualified studies were entered to the research that obtained a minimum score^14^ using this checklist. Moreover, COREQ consists of 32 items, including the researcher's profile, the type of study design (sampling methods, sample size, type of interview and methodology), and the method of analyzing and reporting the results^15^. The minimum score was 23 to be included in the review.

### Data extraction and analysis

The following information was extracted from each paper entered to the study: first author, year of publication, study type, number and mean age of participants, location of the study, type of population surveyed, type of instrument used, and most important findings. The validity of the tool was evaluated by 10 expert faculty members in Mazandaran University of Medical Sciences.

### Ethics statement

This study was approved in Ethic committee of Mazandaran University of Medical Sciences (IR.MAZUMS.REC.1398.453).

## Results

All articles entered into this study were reviewed by two checklists. Overall, the quality of studies was acceptable.

In the present study, 15 articles out of 59 articles were entered after various stages of screening^5^,^8^,^16^^-^^28^. Most of them (86.6%) had cross-sectional designs and were conducted in the USA (73.3%). Furthermore, most of the studies about self-blaming were among patients with cancer, but few studies have been conducted on patients with heart disease, COPD, burns, Major Depressive Disorders (MDD), type 2 diabetes, and irritable bowel syndrome (based on Table 2).

**Table 2.** Characterization of included articles

Author (year)	Study type	Place	Participants (n)	Mean age (SD)	Study tool	Important findings
Malcame (1995)	Cross-sectional	USA	Cancer patients (72)	45.46 (7.49)	BSB and CSB	Psychological distress was an important predictor of self-blame.
Bennett (2005)	Cross-sectional	USA	Breast cancer patients (53)	53 (9.4)	BSB and CSB	Self-blame was an important predictor of anxiety and depression.
Sholomskas (1990)	Cross-sectional	USA	Spinal damage (31)	29	Causal Dimension Scale (CDS)	There was no relationship between self-blame and patient coping behaviors.
Else-Quest (2009)	Cross-sectional	USA	Lung, prostate and breast cancer patients (172)	66.9 (11.18)	The State Shame and Guilt Scale (SSGS)	Self-blame was an important predictor of anxiety, anger and depression.
Bennett (2013)	Cross-sectional	USA	Cardiac patients (129)	64.1 (10.2)	BSB and CSB	BSB was associated with mental distress at the beginning of the trainings.
Zahn	Cross-sectional	England	MDD (132)	32.8 (12.3)	Psycho-pathology-based instrument (AMDP)	Self-blame was high in MDD patients, but it was not the cause of sin.
Glinder (1999)	Cross-sectional	USA	Breast cancer patients (76)	54.8 (9.8)	BSB and CSB	Self-blame was an important predictor of anxiety and depression.
Kiecolt-Glaser (1987)	Cross-sectional	USA	Burn patients (49)	35.87 (13.44)	BSB and CSB	The higher BSB predicted depression and inconsistency.
Friedman (2007)	Cross-sectional	USA	Breast cancer patients (123)	56.2 (11.5)	BSB and CSB	Self-blame was an important predictor of anxiety and depression.
Sheridan (2011)	Qualitative	New Zealand	COPD (29)	-	Interview	Self-blame has increased the sense of need.
Halding (2010)	Qualitative	Norway	COPD (18)	-	Interview	Self-blame was remarkable in these patients.
Phelan (2011)	Cross-sectional	USA	Colorectal cancer (1109)	68.4 (10)	Researcher made	Self-blame was an important predictor of depression.
Friedman (2010)	Cross-sectional	USA	Breast cancer patients (108)	52	BSB and CSB	Self-blame was associated with quality of life.
Beverly (2012)	Qualitative	USA	Type 2 diabetes (34)	59.8 (7.3)	Interview	Self-blame was responsible for poor achievement for medical purposes.
Alisha (2000)	Cross-sectional	Canada	Irritable bowel syndrome (25)	-	BSB and CSB	Self-blame was an important predictor of psychological distress.

The patient ages ranged from 29 to 68.4. Also, most studies used a two-part tool of characterological self-blame (CSB) and behavioral self-blame (BSB) to evaluate self-blaming. Based on the results, self-blaming had a significant relationship with the amount of distress, anxiety, and depression in patients. In other words, self-blaming was associated with mental disorders and patients ’ quality of life.

### Self-blame and distress

Some studies were carried out on cancer patients, cardiac patients and irritable bowel syndrome around the relationship of self-blame and psychological distress (Table 2). Malcarne in his study that was done on 72 cancer patients (near diagnosis) declared that characterological self-blame was related to higher levels of psychological distress in the first of study (time 1) and Time 2 (4 months after) [r(70) = .38, *p* < .001] but behavioral self-blame not related to psychological distress in Time 216. Initial psychological distress was a significant predictor of characterological self-blame (*sr*^2^ = .53, p < .001, β = .74), and the interaction of behavioral and characterological self-blame was significant (*sr*^2^ = .02, *p* < .05, β = .18). Bennett released that BSB in cardiac patients was predictive of baseline levels of anxiety symptoms (β=0.28, *p*<0.01, *sr*^2^=0.06), as well as Time 2 (12 weeks later than cardiac rehabilitation program) symptoms after controlling for baseline levels (β=0.23, *p*< 0.01, *sr*^2^=0.04)^28^. That is, blaming one's behavior for a cardiac event was positively related to experiencing symptoms of anxiety concurrently and 12 weeks later.

### Self-blame with anxiety and depression

Else-Quest concluded that self-blame was negatively correlated with self-esteem (r = •0.26, *p*<0.001), but positively correlated with anxiety (r = 0.55, *p*<0.001), anger (r = 0.53, *p*<0.001) and depressed affect (r = 0.55, p<0.001)^18^. Self-blame also has a mediating role on the effect of perceived stigma on adjustment. The Bennett study on cardiac patients showed that BSB was positively related to baseline depressive symptoms (β=0.38, *p*<0.001, *sr*^2^=0.11), and it predicted symptoms of depression at Time 2 after controlling for baseline levels (β=0.14, *p*<0.05, *sr*^2^=0.01)^28^. Another research on newly diagnosed breast cancer patients declared that, at diagnosis, the regression equation was significant in predicting anxiety and depression symptoms, accounting for 20% of the variance8. Level of distress was predicted by behavioral self-blame; greater behavioral self-blame was predictive of more anxiety and depression symptoms, accounting for 9% unique variance. Characterological self-blame was not significantly associated with affective symptoms. When behavioral (β = .38, *sr*^2^ = .14) and characterological self-blame (β = .28, *sr*^2^ = .08) were entered separately, both were significant predictors of anxiety and depression symptoms^8^.

In the Kiecolt-Glaser study, the burn patients ’ self-blame had a significant relation with depression (r = 0.15, β = 0.32, *p*<0.05), but no significant relation was seen with anxiety^20^. Phelan, in a study on colorectal cancer patients, also concluded that self-blame was significantly associated with depressive symptoms (b = 2.67, *p*<0.001). The perception that other people blame the individual with colorectal cancer for the disease remained non-significant (b = −0.58, p = 0.56)^25^.

### Other related factors

One of the important factors that was assessed by Friedman on breast cancer patients was quality of life^21^. Self-blame, spirituality, and employment status were significant predictors of quality of life, accounting for 47% of the variance (p<0.001). Also the results from the multiple regression analyses show self-blame, self-forgiveness, and spirituality were significant predictors of mood disturbance, accounting for 50% of the variance (p<0.001)^21^. Sholomskas stated that self-blaming had no significant relationship with coping skills in spinal injury patients^17^. Also one concept that was focused in some studies was self-blaming rumination that effects on psychological distress and other related psychological diseases including depression^29^.

## Discussion

There are numerous studies about self-blaming; this is still an important and new subject throughout the world. One important conclusion of this review is that self-blaming is a significant predictor of psychological distress, anxiety, and depression in patients. It is problematic when it appears as self-blaming rumination^30^. Self-blaming rumination is a form of intellectual rumination and negative recurring thinking with the purpose of underestimating oneself. Moreover, researchers have suggested that it is associated with psychological problems such as depression, anxiety, and anger^31^. Self-blaming is a form of inadequate self which is correlated with marital conflicts^32^. Also, it is related to disappointment and indifference about others, that is, they feel apathy about them^33^. Abi-Habib and Luyten found in their research that these kinds of people behaved in a hostile and biased manner against others^34^. They are anxious and hardly in control of their anger towards others, which often leads to conflicts in interpersonal relationships.

So far, most of the studies about this phenomenon have been conducted in samples of US populations. Cancer patients were the most emphasized sample, cardiac and COPD patients were the next grades. Unfortunately, no attention has been paid to this very important issue on patients in Iran. However, Kochaki et al. conducted a study about the mediating role of self-blaming rumination in the relationship between disaffection and marital violence. They stated that self-blaming can't predict marital violence^35^. It needs to consider that the sample population of this study was community samples who have different mental and physical conditions compared to populations of patients with medical diagnosis.

Patients are subjected to suffering from various psychological changes due to possible changes in their organs (because of the process of disease or surgical procedures) and the reductions of energy and activities associated with such illnesses. Changes in their self-concept is one of the most common and destructive changes^36^. Patients with disrupted self-concept tend to blame themselves. It can lead to giving up against the disease in extreme cases, which can have adverse health effects for the patients. Paying attention to this phenomenon will greatly contribute to the health and wellbeing of a community in terms of manpower and medical expenses.

The hopelessness theory of depression proposes that depression is caused by two variables: attribution of negative events to stable and global causes, and other cognitive factors like low self-esteem^37^. CSB attributes occurrence of events to stable aspects of the individual that are not controllable. CSB attributions seem likely to cause helplessness, since individuals believe they are powerless to control the characteristics that lead to negative events^38^. On the other hand, BSB has an indeterminate effect under hopelessness theory, since BSB attributes events to behaviors that can be controlled to produce better outcomes^38^. These theories of attributional style and stress and coping have similar predictions to Janoff-Bulman's BSB/CSB distinction^6^. Depression occurs when individuals feel that they cannot control the future. The CSB/BSB distinction also corresponds to Dweck's distinction between ability and effort attributions^39^. Effort attributions are when individuals assign success or failure to the hard work and other controllable factors, while ability attributions assign outcomes to internal, stable characteristics, like intelligence. Dweck noted that individuals that believe outcomes are uncontrollable are more likely to be debilitated by setbacks, procrastinate or avoid stressors, and show greater stress responses^39^. In short, theorists believe that the type of cause to which events are attributed is a central factor of effectiveness of blame^40^.

Exploratory neuroscientific evidence has found a distinct sign of connectivity abnormality associated with general self-blame. Evidence suggests that major depressive disorder creates vulnerability to depression that lasts years after the cessation of depressive episodes^41^. One of the mechanisms of this “scar theory“ of depression is proposed to be increased likelihood to perform self-blame. Self-blaming biases are present in patients with remitted depression, and these biases are associated with risk of recurrence of MDD^41^. Researchers used functional magnetic resonance imaging (fMRI) to examine brain regions and connections associated with self-blame. Abnormal activation was demonstrated in subgenual cingulate cortex and septal area (SCSR) in currently depressed individuals, but in other situations as well: previously-depressed individuals showed differences in brain activity while feeling guilt compared to always-healthy controls^42^. Also, the amount of connection abnormality in these regions was predictive of depression recurrence^42^. These data suggest that depression episodes change the quality of self-blame, making individuals vulnerable to depression recurrence.

The result of this systematic review reveals the fact that the issue of self-blaming is very important and is also a key for all groups of society, especially patients. Unfortunately, this issue has not been adequately considered in Iran. By emphasizing the use of specific tools to measure self-blaming for patients, some interventions can be initiated to treat these disorders.

Moreover, the practice of using this tool and its necessity can be taught in the universities and especially in nursing schools (due to the high level of nurse-patient relationship). The collection of all these suggestions can be effective over time for the health of patients, as well as their families, which helps to reduce the burden of treatment on the country's side, in addition to promoting the country's health index in the world.

This study had some limitations like other studies. One of the most important was the shortage of articles for more definitive and stable conclusions. Also, no relevant studies were found on patients in Iran that allowed us to compare our findings with other communities. Most of the focus was on cancer patients, so a more accurate comparison between patient populations was not possible. Given only seven databases were used in the study, the possibility exists that certain relevant publications containing self-blame measures were not identified, a common challenge when conducting systematic reviews. Furthermore, given our initial review targeted article abstracts, it is plausible that information about the measures were included in the text of omitted articles.

Therefore, it is recommended that future studies be carried out on well-defined population of patients. Of course, it is important to note that the tool to measure self-blaming has not been validated in Iran. So, it needs to be validated in a Farsi-speaking Persian sample. Now we know that self-blame had significant relation with some important psychological factors like depression and anxiety, so we can design and run some randomized clinical trial studies to assess the exact effects of reducing self-blame on those negative psychological factors.

In summary, a significant relation was reported between self-blaming and the degree of distress, anxiety, and depression in patients in most of the studies. It is recommended to do further studies to evaluate this finding.
